# Cardiovascular Impact of Endovascular Revascularization in Chronic Limb-Threatening Ischemia Versus Claudication: A Contemporary Real-World Analysis

**DOI:** 10.1016/j.jscai.2026.105372

**Published:** 2026-04-23

**Authors:** Sultana Jahan, Ali Awad, Mustafa Al-Mollah, Moaiad Hussein, Nader Alwifati, Khadeeja Sirajuddin, Mariam Chalhoub, Muhammad Burhan, Charles Danish, Aisha Tanveer, Timir K. Paul, M. Chadi Alraies

**Affiliations:** a Department of Medicine, Valley Health System, Las Vegas, Nevada; b Department of Medicine, Detroit Medical Center/Wayne State University, Detroit, Michigan; c Department of Medicine, Corewell Health, Dearborn, Michigan; d Department of Medicine, Norwalk Hospital, Norwalk, Connecticut; e Department of Medicine, Raleigh General Hospital, Beckley, West Virginia; f Department of Medicine, Cardiology Division, Prince Sultan Cardiac Center, Riyadh, Saudi Arabia; g Department of Medicine, Case Western Reserve University, University Hospitals Cleveland Medical Center, Cleveland, Ohio; h Department of Medicine, Rawalpindi Medical University, Rawalpindi, Pakistan; i Department of Cardiovascular Sciences, University of Tennessee–Nashville, Ascension St. Thomas Hospital, Nashville, Tennessee; j Division of Cardiovascular Medicine, Department of Medicine, Detroit Heart Hospital, Detroit, Michigan

**Keywords:** chronic limb-threatening ischemia, endovascular revascularization, peripheral artery disease

## Abstract

**Background:**

Chronic limb-threatening ischemia (CLTI) represents the most severe form of peripheral artery disease, yet long-term cardiovascular outcomes after endovascular revascularization compared with claudication remain poorly defined.

**Methods:**

Using the TriNetX global research network (114 health care organizations), adults undergoing endovascular lower-extremity revascularization for CLTI or intermittent claudication were identified. Propensity score matching for demographics, comorbidities, and cardiovascular medications yielded 3727 matched pairs. Major adverse cardiovascular events (all-cause mortality, myocardial infarction, or stroke) and secondary outcomes were assessed at 1, 3, and 5 years.

**Results:**

At 5 years, patients with CLTI experienced a significantly higher risk of major adverse cardiovascular events compared with those with claudication (34.9% vs 30.7%; *P* < .001), along with greater all-cause mortality (18.7% vs 15.6%; *P* < .001), heart failure hospitalization (15.0% vs 13.0%; *P* = .001), major bleeding (15.1% vs 13.3%; *P* = .002), and acute kidney injury (26.4% vs 22.2%; *P* < .001). Rates of stroke or transient ischemic attack were similar between groups, whereas repeat revascularization was lower among patients with CLTI (45.9% vs 51.2%; *P* = .002). These findings were consistent at 1- and 3-year follow-up.

**Conclusions:**

Chronic limb-threatening ischemia patients undergoing endovascular revascularization experience significantly worse short- and long-term cardiovascular outcomes compared with claudication, underscoring the need for intensified cardiovascular risk stratification and secondary prevention in this high-risk population following peripheral intervention.

## Introduction

Peripheral arterial disease (PAD) ranks among the 5 most prevalent cardiovascular diseases globally and is rapidly increasing in burden, with prevalence expected to more than double over the next 25 years.^1^, ^2^, ^3^ Symptomatic lower extremity PAD exists along a continuum ranging from intermittent claudication to chronic limb-threatening ischemia (CLTI).^4^, ^5^, ^6^ CLTI represents the end stage of PAD and is characterized by severe atherosclerosis, ischemic rest pain, and/or tissue loss, accounting for most lower extremity amputations worldwide.^7^^,^^8^

At the population level, even asymptomatic PAD confers cardiovascular risk comparable to prior myocardial infarction and is, therefore, managed as a coronary artery disease risk equivalent, warranting aggressive secondary prevention.^9^^,^^10^ Endovascular revascularization has become the predominant revascularization strategy for PAD across the spectrum of disease severity, driven by advances in device technology, expanding indications, and lower procedural morbidity compared with surgical bypass.^11^ Although endovascular therapy improves limb perfusion and limb-related outcomes, PAD remains a systemic atherosclerotic disease, and cardiovascular events continue to be a major determinant of long-term prognosis, particularly in patients with CLTI.^10^ Prior observational studies and registries have demonstrated higher rates of mortality and adverse events among CLTI patients; however, these differences are often attenuated after adjustment for baseline comorbidities, suggesting that competing cardiovascular risk and treatment selection play a substantial role in observed outcomes.^12^

Importantly, existing studies have primarily focused on short-term outcomes, limb-specific end points, or comparisons between revascularization modalities, rather than isolating the prognostic impact of disease severity among patients treated with the same contemporary intervention. Data directly comparing long-term cardiovascular outcomes between CLTI and claudication patients undergoing endovascular revascularization, after balancing measured baseline risk, remain limited.^11^, ^12^, ^13^ Presentation with CLTI confers excess cardiovascular risk despite receipt of endovascular therapy, and adjustment for comorbid burden is, therefore, incompletely defined, particularly over extended follow-up.

Accordingly, we sought to evaluate short- and long-term cardiovascular outcomes in patients with CLTI versus intermittent claudication who underwent endovascular lower-extremity revascularization using a large, real-world electronic health record network. Propensity score matching was used to balance demographic characteristics, comorbidities, and cardiovascular medication use. Major adverse cardiovascular events (MACE) and secondary outcomes at 1, 3, and 5 years were compared to assess the prognostic implications of baseline disease severity following peripheral endovascular intervention.

## Methods

### Study population

This retrospective cohort study utilized deidentified electronic health record data from the TriNetX Research Network, a global federated health research database comprising electronic medical records from 114 participating health care organizations across the United States. Adult patients (≥18 years old) with a diagnosis of peripheral artery disease (PAD) who underwent endovascular lower extremity revascularization were identified using standardized diagnostic and procedural codes. Patients were identified from December 2005 through December 2025, with data extraction performed on December 24, 2025. Inclusion required at least 1 diagnostic code consistent with PAD involving native arteries or bypass grafts of the lower extremities and at least 1 qualifying endovascular revascularization procedure involving the iliac, femoral, popliteal, or infrapopliteal arterial segments. Detailed diagnostic and procedural codes used for cohort identification are provided in Supplemental Table S1.

Two cohorts were defined based on clinical presentation at the time of revascularization. The CLTI cohort included patients with PAD complicated by ischemic rest pain, ischemic ulceration, or gangrene. The claudication cohort included patients with intermittent claudication without evidence of rest pain or tissue loss. To ensure appropriate temporal association, qualifying diagnostic codes were required to occur on or before the index endovascular procedure, and patients meeting criteria for both cohorts were classified according to the most severe clinical presentation at the time of intervention.

Patients with a history of major lower extremity amputation were excluded to ensure a uniform at-risk limb population and to minimize confounding related to advanced irreversible limb loss. Patients with prior surgical lower-extremity bypass revascularization were also excluded to create a homogeneous cohort undergoing index endovascular intervention, as prior bypass surgery may substantially alter vascular anatomy, subsequent reintervention patterns, and long-term limb and survival outcomes. Patients with incomplete demographic data or no follow-up after the index event were excluded from the final analysis.

Outcomes were assessed following the index endovascular revascularization. Comprehensive outcome definitions are provided in Supplemental Tables S1 and S2.

The index event was defined as the first documented endovascular lower-extremity revascularization meeting cohort criteria, and patients whose index event occurred more than 20 years prior to data extraction were excluded in accordance with TriNetX analytic standards.

### Data collection and outcomes

Demographic characteristics, diagnoses, procedures, medications, and encounter data were extracted using the TriNetX analytics platform and standardized across institutions using established coding systems, including ICD-10-CM, ICD-10-PCS, CPT, and HL7 visit types. Clinical outcomes were assessed over 1-, 3-, and 5-year follow-up periods, beginning 1 day after the index event. Before matching, the CLTI cohort comprised 56,859 patients, and the claudication cohort comprised 3727 patients. After propensity score matching, 3727 patients remained in each cohort, with standardized mean differences <0.1 across all baseline covariates, indicating adequate balance.

Major bleeding in the secondary outcome was defined using diagnostic codes corresponding to clinically significant bleeding events requiring hospitalization. These included intracranial hemorrhage, intracerebral hemorrhage, subarachnoid hemorrhage, gastrointestinal bleeding, hematemesis, melena, acute peptic ulcer with hemorrhage, intraoperative hemorrhage or hematoma, postprocedural hemorrhage, and other bleeding events necessitating inpatient-level care. This definition is consistent with prior administrative database studies evaluating major bleeding outcomes. Detailed diagnostic and procedural codes used for outcome ascertainment are provided in Supplemental Tables S1 and S2.

### Statistical analysis

To reduce baseline confounding, 1:1 propensity score matching was performed using a nearest neighbor algorithm. Matching variables included age, sex, race, cardiovascular comorbidities (coronary artery disease, heart failure, hypertension, diabetes mellitus, chronic kidney disease, and cerebrovascular disease), and baseline cardiovascular medication use. Balance between cohorts was evaluated using standardized mean differences, with values <0.1 indicating acceptable balance. Outcomes were compared using risk analyses to generate hazard ratios (HR) with corresponding 95% CIs. Time-to-event outcomes were assessed using Kaplan-Meier survival analysis with log-rank testing. All analyses were conducted within the TriNetX platform using real-time statistical computation, and a 2-sided *P* value <.05 was considered statistically significant.

### Ethical considerations

Because this study used deidentified aggregate data obtained from the TriNetX Research Network, no institutional review board approval or patient consent was required, in accordance with 45 CFR §46 and institutional policies governing exempt research.

## Results

### Baseline characteristics

Among 60,586 adult patients with PAD undergoing endovascular lower-extremity revascularization who met inclusion criteria, 56,859 patients (93.9%) presented with CLTI, while 3727 patients (6.1%) presented with intermittent claudication. This imbalance reflects the restriction of the cohort to patients undergoing endovascular lower-extremity revascularization, a treatment more commonly performed for CLTI than for intermittent claudication in contemporary practice. Before matching, baseline demographic and clinical characteristics differed significantly between groups, reflecting differences in disease severity and comorbidity burden (Table 1). Propensity scores were calculated using a multivariable logistic regression model that included age, sex, race, coronary artery disease, heart failure, hypertension, diabetes mellitus, chronic kidney disease, and prior cerebrovascular disease. Dual antiplatelet therapy, defined as aspirin in combination with a P2Y12 inhibitor (clopidogrel, prasugrel, or ticagrelor), was observed in 37,801 patients with CLTI undergoing endovascular intervention and in 2362 patients with claudication undergoing endovascular intervention at baseline. After 1:1 propensity score matching, 3727 matched pairs were included in the final analysis. Standardized mean differences were <0.1 across all baseline covariates, indicating adequate balance between cohorts. In the matched cohort, most baseline characteristics were similar between groups (Table 1). Rates of coronary artery disease, heart failure, hypertension, diabetes mellitus, chronic kidney disease, and prior cerebrovascular disease did not differ significantly between patients with CLTI and those with claudication after propensity score matching.

**Table 1:** Baseline characteristics before and after propensity score matching.

	Before propensity matching				After propensity matching		
	CLTI + endovascular (n = 56,859)	Claudication + endovascular (n = 3727)	*P* value	Standard difference	CLTI + endovascular (n = 3727)	Claudication + endovascular (n = 3727)	*P* value
Age, y							
Current age	74.4	73.8	.003	0.051	74.1	73.8	.374
Age at index	69.2	67.3	<.001	0.177	67.5	67.3	.406
Sex							
Female	23,991 (42.2)	1437 (38.6)	<.001	0.074	1436 (38.5)	1437 (38.6)	.981
Male	32,862 (57.8)	2289 (61.4)	<.001	0.074	2291 (61.5)	2289 (61.4)	.962
Race							
White	41,489 (73.0)	2955 (79.3)	<.001	0.149	2982 (80.0)	2955 (79.3)	.437
Black or African American	10,229 (18.0)	469 (12.6)	<.001	0.151	461 (12.4)	469 (12.6)	.779
Comorbidities							
Essential (primary) hypertension	44,160 (77.7)	2710 (72.7)	<.001	0.115	2697 (72.4)	2710 (72.7)	.736
Diabetes mellitus	30,578 (53.8)	1405 (37.7)	<.001	0.327	1362 (36.5)	1405 (37.7)	.303
Hyperlipidemia, unspecified	35,301 (62.1)	2182 (58.5)	<.001	0.072	2162 (58.0)	2182 (58.5)	.639
Other hyperlipidemia	12,049 (21.2)	911 (24.4)	<.001	0.078	875 (23.5)	911 (24.4)	.329
Atherosclerotic heart disease of native coronary artery	27,933 (49.1)	1783 (47.8)	.127	0.026	1741 (46.7)	1783 (47.8)	.33
Heart failure	28,950 (50.9)	1837 (49.3)	.054	0.033	1791 (48.1)	1837 (49.3)	.286
CKD	16,806 (29.6)	711 (19.1)	<.001	0.246	670 (18.0)	711 (19.1)	.222
Chronic ischemic heart disease	18,326 (32.2)	755 (20.3)	<.001	0.275	764 (20.5)	755 (20.3)	.796
Cerebral infarction	10,283 (18.1)	574 (15.4)	<.001	0.072	560 (15.0)	574 (15.4)	.652
Old myocardial infarction	6774 (11.9)	321 (8.6)	<.001	0.109	336 (9.0)	321 (8.6)	.54
Atrial fibrillation and flutter	11,878 (20.9)	505 (13.5)	<.001	0.195	490 (13.1)	505 (13.5)	.609
Nicotine dependence	20,374 (35.8)	1438 (38.6)	.001	0.057	1409 (37.8)	1438 (38.6)	.489
Medication							
Aspirin	35,653 (62.7)	2152 (57.7)	<.001	0.102	2109 (56.6)	2152 (57.7)	.314
Clopidogrel	19,553 (34.4)	1362 (36.5)	.007	0.045	1325 (35.6)	1362 (36.5)	.372
Prasugrel	821 (1.4)	78 (2.1)	.002	0.049	67 (1.8)	78 (2.1)	.356
Ticagrelor	1446 (2.5)	96 (2.6)	.903	0.002	96 (2.6)	96 (2.6)	1
Cangrelor	83 (0.1)	10 (0.3)	.065	0.027	10 (0.3)	10 (0.3)	1
Atorvastatin	28,618 (50.3)	1701 (45.6)	<.001	0.094	1655 (44.4)	1701 (45.6)	.284
Rosuvastatin	8688 (15.3)	622 (16.7)	.021	0.038	633 (17.0)	622 (16.7)	.733
ACE inhibitors	23,331 (41.0)	1433 (38.4)	.002	0.053	1381 (37.1)	1433 (38.4)	.214
Angiotensin II receptor blockers	15,342 (27.0)	907 (24.3)	<.001	0.061	893 (24.0)	907 (24.3)	.705
Metoprolol	24,647 (43.3)	1430 (38.4)	<.001	0.101	1414 (37.9)	1430 (38.4)	.703
Carvedilol	10,181 (17.9)	512 (13.7)	<.001	0.114	482 (12.9)	512 (13.7)	.307
Bisoprolol	461 (0.8)	35 (0.9)	.4	0.014	31 (0.8)	35 (0.9)	.621
Warfarin	5579 (9.8)	268 (7.2)	<.001	0.094	266 (7.1)	268 (7.2)	.928
Apixaban	6704 (11.8)	260 (7.0)	<.001	0.166	266 (7.1)	260 (7.0)	.786
Rivaroxaban	3220 (5.7)	176 (4.7)	.016	0.042	174 (4.7)	176 (4.7)	.913
Edoxaban	13 (0.0)	10 (0.3)	<.001	0.064	0 (0.0)	10 (0.3)	.002
Dabigatran etexilate	425 (0.7)	34 (0.9)	.261	0.018	40 (1.1)	34 (0.9)	.483
SGLT2 inhibitors	4593 (8.1)	218 (5.8)	<.001	0.088	229 (6.1)	218 (5.8)	.592
GLP-1 receptor agonists	3393 (6.0)	166 (4.5)	<.001	0.068	178 (4.8)	166 (4.5)	.508

Values are n (%).

CKD, chronic kidney disease; CLTI, Chronic limb-threatening ischemia.

### Outcomes

Outcomes were assessed over 3 cumulative follow-up windows from the index endovascular revascularization: 1 year, 3 years, and 5 years. The primary outcome was MACE. Secondary outcomes included all-cause mortality, myocardial infarction, stroke or transient ischemic attack (TIA), heart failure hospitalization, major bleeding, acute kidney injury (AKI), and repeat lower-extremity revascularization.

**Figure 1:**
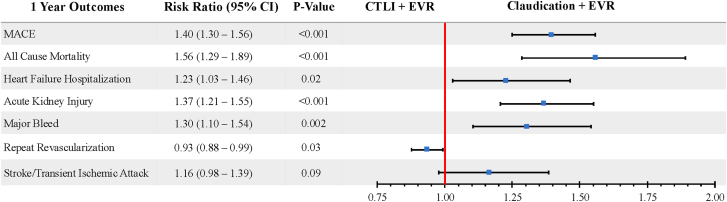
**Adjusted 1-year clinical outcomes comparing chronic limb-threatening ischemia (CLTI) and claudication following endovascular revascularization (EVR) (forest plot).** MACE, major adverse cardiovascular events.

At 1 year, patients with CLTI demonstrated higher cumulative event rates compared with patients treated for claudication. The incidence of MACE was 17.3% versus 12.4% (risk ratio [RR], 1.40; 95% CI 1.29-1.64; *P* < .001), and all-cause mortality occurred in 6.7% versus 4.3% (RR 1.56, 95% CI, 1.31-1.95; *P* < .001). Heart failure hospitalization (6.9% vs 5.7%; RR, 1.23; 95% CI, 1.05-1.51; *P* = .014), major bleeding (7.9% vs 6.1%; RR, 1.30; 95% CI 1.12-1.59; *P* = .001), and AKI (13.7% vs 10.0%; RR, 1.34; 95% CI, 1.24-1.63; *P* < .001) were also significantly more frequent among patients with CLTI. Rates of stroke/TIA (6.9% vs 5.9%; RR, 1.16; 95% CI, 1.00-1.43; *P* = .056) and repeat revascularization (33.5% vs 35.9%; RR, 0.93; 95% CI, 0.87-1.01; *P* = .087) were similar between groups (Figure 1, Supplemental Table S3).

**Figure 2:**
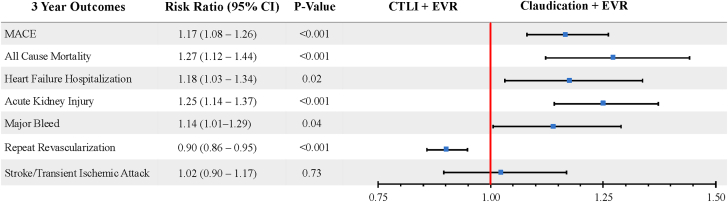
**Adjusted 3-year clinical outcomes comparing chronic limb-threatening ischemia (CLTI) and claudication following endovascular revascularization (EVR) (forest plot).** MACE, major adverse cardiovascular events.

By 3 years, separation in cumulative event incidence persisted. MACE occurred in 27.7% of CLTI patients compared with 23.7% of those with claudication (RR, 1.17; 95% CI, 1.14-1.37; *P* < .001), while mortality rates were 13.2% versus 10.4% (RR, 1.27; 95% CI, 1.18-1.54; *P* < .001). Heart failure hospitalization (11.9% vs 10.1%; RR, 1.17; *P* = .002), major bleeding (12.6% vs 11.0%; HR, 1.20; *P* = .008), and AKI (21.9% vs 17.5%; RR, 1.25; *P* < .001) remained significantly higher among CLTI patients. Stroke/TIA rates were comparable between groups (10.5% vs 10.3%; RR, 1.02; *P* = .334), whereas repeat revascularization occurred less frequently in the CLTI cohort (43.0% vs 47.6%; RR, 0.90; *P* = .006) (Figure 2, Supplemental Table S4).

**Figure 3:**
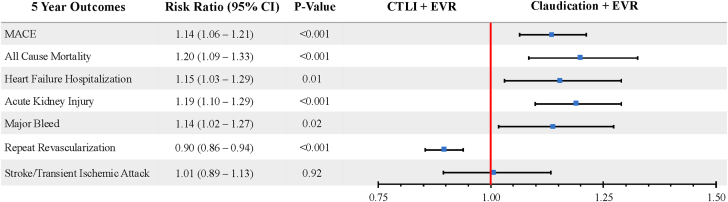
**Adjusted 5-year clinical outcomes comparing chronic limb-threatening ischemia (CLTI) and claudication following endovascular revascularization (EVR) (forest plot).** MACE, major adverse cardiovascular events.

At 5 years, long-term patterns were consistent. MACE occurred in 34.9% of patients with CLTI versus 30.7% with claudication (RR, 1.13; 95% CI, 1.13-1.33; *P* < .001), and mortality rates were 18.7% vs 15.6% (RR, 1.19; 95% CI, 1.15-1.44; *P* < .001). Heart failure hospitalization (15.0% vs 13.0%; RR, 1.15; *P* = .001), major bleeding (15.1% vs 13.3%; RR, 1.14; *P* = .002), and AKI (26.4% vs 22.2%; RR, 1.19; *P* < .001) remained elevated in the CLTI cohort. Stroke/TIA incidence remained similar (12.9% vs 12.8%; RR, 1.01; *P* = .329), whereas repeat revascularization continued to occur less frequently among CLTI patients (45.9% vs 51.2%; RR, 0.90; *P* = .002) (Figure 3, Supplemental Table S5). Survival analysis demonstrated no significant difference between the 2 groups (HR, 1.065; 95% CI, 0.938-1.209; log-rank *P* = .329) (Supplemental Figure S1).

## Discussion

In this large real-world analysis of patients undergoing endovascular lower-extremity revascularization, CLTI was associated with a significantly higher risk of MACE compared with intermittent claudication across 1, 3, and 5 years of follow-up. Despite propensity matching for demographic characteristics, major cardiovascular comorbidities, and baseline cardiovascular medication use, patients with CLTI experienced higher rates of all-cause mortality, heart failure hospitalization, AKI, and major bleeding. In contrast, rates of stroke or TIA were similar between groups, whereas repeat revascularization occurred less frequently among patients with CLTI at 3 and 5 years. The observed difference in MACE was primarily reflected by higher all-cause mortality (Central Illustration).

**Central Illustration:**
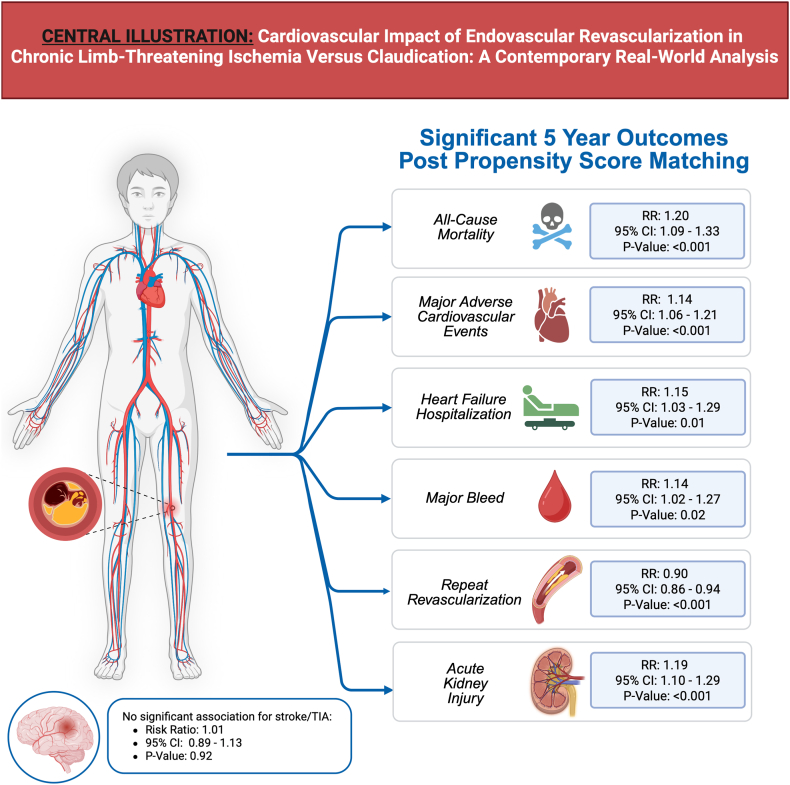
**Long-term cardiovascular impact of endovascularlower-extremityrevascularization in chroniclimb-threateningischemia versus claudication.** RR, risk ratio; TIA, transient ischemic attack.

The marked difference in cohort sizes prior to matching reflects contemporary treatment patterns. Patients with intermittent claudication are often managed initially with supervised exercise therapy and optimized medical therapy, with revascularization reserved for refractory lifestyle-limiting symptoms refractory to conservative treatment. In contrast, CLTI represents a limb-threatening condition for which revascularization is strongly indicated and more frequently performed. Accordingly, limiting the cohort to patients who underwent endovascular intervention resulted in a higher proportion of CLTI presentations.

After matching, the persistent elevation in mortality and adverse cardiovascular outcomes observed in patients with CLTI suggests that revascularization alone may be insufficient to offset the broader systemic risk profile of this population.^11^ Although baseline characteristics were balanced through propensity matching, CLTI may capture elements of disease severity not fully represented by conventional comorbidity adjustment, including features associated with more diffuse atherosclerotic burden, reduced physiologic reserve, or other unmeasured clinical factors.^11^^,^^12^ In this context, CLTI may function less as a purely anatomic diagnosis and more as a clinical marker of heightened systemic risk following peripheral intervention.^14^

The findings of the present study also highlight the importance of comprehensive medical therapy in patients with PAD undergoing revascularization. Baseline cardiovascular medication use, including antiplatelet agents, statins, renin-angiotensin system inhibitors, and beta-blockers, was balanced between cohorts after propensity score matching (Table 1), suggesting that differences in outcomes were not primarily driven by measured differences in pharmacologic therapy. Nonetheless, the persistence of higher rates of MACE among patients with CLTI underscores that endovascular revascularization alone may be insufficient to mitigate the systemic cardiovascular risk associated with advanced PAD.^15^ These observations reinforce the central role of aggressive guideline-directed medical therapy and comprehensive cardiovascular risk reduction strategies alongside limb-directed intervention in this high-risk population.^6^^,^^12^

Our findings are broadly consistent with prior observational studies demonstrating worse unadjusted outcomes among patients with CLTI, although results across registries have varied after risk adjustment.^11^^,^^13^^,^^16^^,^^17^ For example, the Excellence in PAD registry demonstrated higher crude mortality among patients with CLTI; however, mortality was comparable between CLTI and claudication patients after matching.^12^ In contrast, our analysis, which restricts the study population to patients undergoing endovascular lower-extremity revascularization and applies propensity score matching for demographic characteristics, major cardiovascular comorbidities, and baseline medication use, demonstrates that CLTI status remains associated with worse long-term outcomes despite adjustment. These differences likely reflect heterogeneity in study populations, outcome definitions, and duration of follow-up, rather than true disagreement regarding the underlying cardiovascular risk associated with CLTI.^12^

Randomized trial data further support the persistence of adverse systemic outcomes in patients with advanced limb ischemia despite revascularization.^16^, ^17^, ^18^, ^19^ In the Best Endovascular versus Best Surgical Therapy in Patients with Critical Limb Ischemia trial, all-cause mortality remained high during follow-up regardless of revascularization strategy, underscoring that limb-directed intervention alone does not substantially mitigate long-term survival risk.^17^ Similar findings were reported in the Bypass versus Angioplasty in Severe Ischaemia of the Leg trial, which demonstrated poor long-term survival among patients with advanced limb ischemia despite revascularization.^18^ Notably, much of the CLTI literature has prioritized limb-specific end points, such as major adverse limb events, with comparatively fewer studies focusing on MACE.^11^, ^12^, ^13^, ^14^^,^^17^, ^18^, ^19^ In this context, the present analysis, which restricts the cohort to endovascularly treated patients and applies propensity score matching at baseline, demonstrates that CLTI status remains associated with worse long-term cardiovascular outcomes despite adjustment, complementing prior trial and registry data rather than conflicting with them.

Heart failure hospitalization occurred more frequently among patients with CLTI. This may reflect a higher prevalence of subclinical or established cardiac dysfunction, greater susceptibility to physiologic stress, or limited cardiovascular reserve in patients with advanced PAD.^20^^,^^21^ Although mechanistic pathways cannot be directly assessed in this analysis, the increased incidence of heart failure admissions highlights the systemic cardiovascular burden associated with CLTI beyond limb ischemia alone.^20^^,^^21^

Acute kidney injury was more frequently observed among patients with CLTI, but this finding should be interpreted cautiously, given the absence of event-level clinical detail. The higher incidence of AKI may reflect greater baseline vulnerability in patients with advanced PAD, including higher comorbidity burden, exposure to procedural stressors, or hemodynamic instability, although these factors cannot be directly assessed in this analysis.

Major bleeding events were more frequent among patients with CLTI. Emerging evidence suggests that CLTI represents a residual and independent bleeding risk phenotype following endovascular therapy, even after accounting for traditional high bleeding risk features and antithrombotic regimen.^22^, ^23^, ^24^ Patients with CLTI often exhibit a clustering of bleeding-prone characteristics, including chronic kidney disease, anemia, heart failure, and frailty, many of which are incorporated into established bleeding risk frameworks.^22^, ^23^, ^24^ Beyond comorbidity burden, advanced PAD has been associated with systemic vascular remodeling, including medial arterial calcification and chronic inflammatory activation, processes that may contribute to vascular fragility and impaired hemostatic balance.^22^ When considered alongside the frequent need for intensive antithrombotic therapy after revascularization, these features provide clinical context for the heightened bleeding risk observed in patients with CLTI.^22^, ^23^, ^24^

In contrast, the incidence of stroke or TIA did not differ significantly between groups. This suggests that once PAD becomes clinically manifest, cerebrovascular risk may already be substantially elevated across disease stages, and progression to CLTI may not confer additional incremental risk.^16^ This pattern has been observed in other PAD cohorts and supports the concept of PAD as a high-risk cerebrovascular condition irrespective of limb ischemia severity.^17^

Repeat revascularization occurred slightly less frequently among CLTI patients. Although this finding may appear counterintuitive given the greater disease severity associated with CLTI, several factors may explain this pattern. Patients with CLTI experienced substantially higher mortality during follow-up, introducing a competing risk that may reduce the opportunity for subsequent revascularization procedures. In addition, advanced limb ischemia is frequently associated with diffuse distal disease and limited revascularization targets, which may reduce procedural durability and lead clinicians to favor alternative management strategies such as wound care, amputation, or conservative management rather than repeated interventions.^12^^,^^15^ In contrast, patients with intermittent claudication often undergo repeat procedures aimed at improving functional capacity and quality of life, which may explain the higher observed rates of repeat revascularization in this cohort.^12^^,^^15^ Accordingly, lower repeat revascularization rates in CLTI should not be interpreted as improved procedural durability but rather as part of a broader pattern of adverse systemic outcomes.

Taken together, these findings indicate that CLTI identifies a subgroup of patients with PAD who remain at persistently elevated cardiovascular risk despite contemporary endovascular revascularization and adjustment for measured comorbidities.^25^, ^26^, ^27^, ^28^ Although limb-directed interventions remain critical for symptom relief and limb preservation, CLTI status is associated with sustained systemic cardiovascular risk beyond the treated vascular territory.

This study has several important strengths, including the use of a large multiinstitutional real-world data set and the application of propensity score matching to achieve balanced baseline characteristics between groups, thereby reducing potential confounding. Nevertheless, important limitations should be acknowledged. As an observational analysis based on electronic health record data, residual confounding from unmeasured variables cannot be excluded, and clinical details regarding procedural characteristics, physiologic parameters, medication dosing, and event severity were not available. Furthermore, outcomes were identified using diagnostic codes, which may be subject to misclassification and limit mechanistic interpretation. Despite these limitations, the findings have important clinical implications, highlighting that patients with CLTI remain at substantial long-term cardiovascular risk even after successful limb-directed revascularization. These results support the need for intensified secondary prevention, careful bleeding risk assessment, and coordinated multidisciplinary follow-up that extends beyond limb salvage alone. Future studies should focus on identifying modifiable drivers of excess cardiovascular risk in CLTI, evaluating optimized antithrombotic and cardioprotective strategies, and more systematically incorporating cardiovascular end points into clinical trials and longitudinal investigations of advanced PAD.

## Conclusion

In this large contemporary real-world analysis of patients undergoing endovascular lower-extremity revascularization, CLTI was independently associated with a persistently higher risk of MACE compared with intermittent claudication across short- and long-term follow-up. These findings suggest that CLTI represents more than an anatomic manifestation of advanced PAD and serves as a clinical marker of heightened systemic cardiovascular vulnerability that is not fully attenuated by limb-directed revascularization. The persistence of adverse cardiovascular outcomes despite contemporary endovascular therapy underscores the limitations of revascularization alone in mitigating the overall cardiovascular risk burden in this population. Patients with CLTI may require intensified secondary prevention strategies, comprehensive cardiovascular risk optimization, and multidisciplinary longitudinal care extending beyond procedural success to address their substantial residual risk.

## CRediT authorship contribution statement

**Sultana Jahan:** Conceptualization, Data curation, Investigation, Methodology, Project administration, Writing – original draft. **Ali Awad:** Data curation, Formal analysis, Investigation, Methodology, Resources, Software, Writing – original draft. **Mustafa Al-Mollah:** Investigation, Methodology, Validation, Visualization, Writing – original draft. **Moaiad Hussein:** Investigation, Methodology, Visualization, Writing – original draft. **Nader Alwifati:** Investigation, Resources, Validation, Visualization, Writing – original draft. **Khadeeja Sirajuddin:** Resources, Visualization, Writing – original draft. **Mariam Chalhoub:** Software, Visualization, Writing – original draft. **Muhammad Burhan:** Validation, Writing – review & editing. **Charles Danish:** Visualization, Writing – review & editing. **Aisha Tanveer:** Visualization, Writing – review & editing. **Timir K. Paul:** Supervision, Validation, Writing – review & editing. **M. Chadi Alraies:** Project administration, Supervision, Writing – review & editing.

## References

[1] Horváth L., Németh N., Fehér G., Kívés Z., Endrei D., Boncz I. (2022). Epidemiology of peripheral artery disease: narrative review. Life (Basel).

[2] Yan C., Chen J., Xu X., Wei H., Li J. (2025). Global burden of peripheral arterial disease (1990–2021), global burden trends and the impact of blood lead on peripheral arterial disease: a multidimensional analysis based on NHANES, GBD, and Mendelian randomization. J Transl Med.

[3] Deng L., Du C., Liu L., Wang Y. (2025). Forecasting the global burden of peripheral artery disease from 2021 to 2050: a population-based study. Research (Wash D C).

[4] Hardman R.L., Jazaeri O., Yi J., Smith M., Gupta R. (2014). Overview of classification systems in peripheral artery disease. Semin Intervent Radiol.

[5] Aboyans V., Sevestre M.A., Désormais I., Lacroix P., Fowkes G., Criqui M.H. (2018). Épidémiologie de l’artériopathie des membres inférieurs. Presse Med.

[6] Gornik H.L., Aronow H.D., Goodney P.P. (2024). 2024 ACC/AHA/AACVPR/APMA/ABC/SCAI/SVM/SVN/SVS/SIR/VESS guideline for the management of lower extremity peripheral artery disease: a report of the American College of Cardiology/American Heart Association Joint Committee on Clinical Practice Guidelines. Circulation.

[7] Daher G., Upadhyay S., Li J. (2025). Chronic limb-threatening ischemia: a comprehensive review paper. Interv Cardiol Clin.

[8] Meffen A., Rutherford M.J., Sayers R.D., Houghton J.S.M., Bradbury N., Gray L.J. (2025). Regional variation in non-traumatic major lower limb amputation in England: observational study of linked primary and secondary care data. BJS Open.

[9] Golomb B.A., Dang T.T., Criqui M.H. (2006). Peripheral arterial disease: morbidity and mortality implications. Circulation.

[10] Subherwal S., Patel M.R., Kober L. (2015). Peripheral artery disease is a coronary heart disease risk equivalent among both men and women: results from a nationwide study. Eur J Prev Cardiol.

[11] Thukkani A.K., Kinlay S. (2015). Endovascular intervention for peripheral artery disease. Circ Res.

[12] Patel K., Liu Y., Etaee F. (2021). Differences between patients with intermittent claudication and critical limb ischemia undergoing endovascular intervention: insights from the Excellence in Peripheral Artery Disease registry. Circ Cardiovasc Interv.

[13] Turner J.T., Patel A., Jeng M. (2022). Outcome of claudication patients treated with endovascular intervention. J Vasc Surg.

[14] Vemulapalli S., Doros G., Weissler E.H. (2025). Prediction of 1-year major adverse cardiovascular events in chronic limb threatening ischemia. JACC Adv.

[15] Beckman J.A., Schneider P.A., Conte M.S. (2021). Advances in revascularization for peripheral artery disease: revascularization in PAD. Circ Res.

[16] Kinlay S. (2025). Revascularization for chronic limb-threatening ischemia—synthesizing inconsistency. J Soc Cardiovasc Angiogr Interv.

[17] Farber A., Menard M.T., Conte M.S. (2022). Surgery or endovascular therapy for chronic limb-threatening ischemia. N Engl J Med.

[18] Adam D.J., Beard J.D., Cleveland T. (2005). Bypass versus angioplasty in severe ischaemia of the leg (BASIL): multicentre, randomised controlled trial. Lancet.

[19] Chu D., Zheng X., Mao J., Ramsey L., Mukherjee D. (2024). Comparison of endovascular therapies for chronic limb-threatening ischemia and claudication. J Vasc Surg.

[20] Inoue K., Morisaki K., Matsuda D. (2025). Influence of heart failure on the clinical outcomes of patients with chronic limb-threatening ischemia after infrainguinal revascularization. J Vasc Surg.

[21] Fukino K., Ueshima D., Yamaguchi T. (2024). Prognostic impact of reduced left ventricular ejection fraction after endovascular therapy for lower extremities. Circ J.

[22] Hundito A., Wells N., Tuttle M.R. (2024). The incidence and significance of bleeding in patients with advanced peripheral arterial disease. J Vasc Surg.

[23] Tokuda T., Yoshioka N., Koyama A., Yamada T., Shimamura K., Nishikawa R. (2024). Chronic limb-threatening ischemia is a residual bleeding risk factor among patients with lower extremity artery disease. J Atheroscler Thromb.

[24] Hashimoto R., Numasawa Y., Yokokura S. (2021). Prevalence of the Academic Research Consortium high bleeding risk criteria in patients undergoing endovascular therapy for peripheral artery disease in lower extremities. Heart Vessels.

[25] Azuma N., Takahara M., Kodama A. (2019). Predictive model for mortality risk including the wound, ischemia, foot infection classification in patients undergoing revascularization for critical limb ischemia. Circ Cardiovasc Interv.

[26] Foley K.M., Kennedy K.F., Lima F.V. (2024). Treatment variability among patients hospitalized for chronic limb-threatening ischemia: an analysis of the 2016 to 2018 US National Inpatient Sample. J Am Heart Assoc.

[27] Takada T., Shibahashi E., Hasegawa S. (2025). Cardiovascular prognosis in limb ischemia patients with heart failure and systolic dysfunction following major amputation. Am J Cardiol.

[28] Mohamedali A., Kiwan G., Kim T. (2022). Reinterventions in patients with claudication and chronic limb threatening ischemia. Ann Vasc Surg.

