# Supplementary material for: Cardiovascular Impact of Endovascular Revascularization in Chronic Limb-Threatening Ischemia Versus Claudication: A Contemporary Real-World Analysis
Source: J Soc Cardiovasc Angiogr Interv. 2026 Apr 23;5(6):105372. doi: 10.1016/j.jscai.2026.105372 (PMC13404045; doi:10.1016/j.jscai.2026.105372)
Supplement: Supplementary Material [file mmc1.docx]

# Supplementary Appendix

**Supplementary Figure S1.** Survival Analysis – All-cause mortality


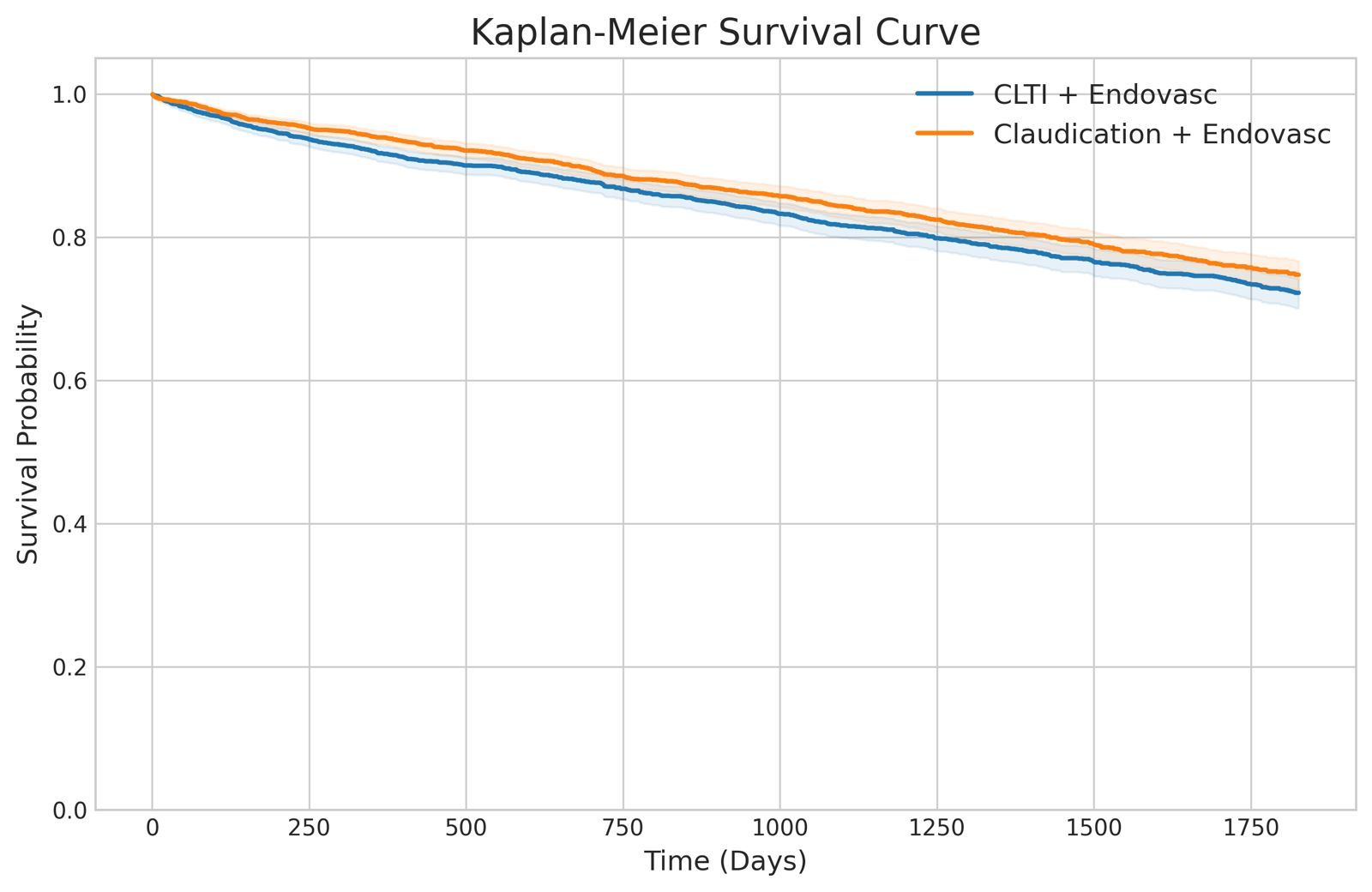


|  | **Kaplan - Meier survival analysis** | | | | | | | | | |
| --- | --- | --- | --- | --- | --- | --- | --- | --- | --- | --- |
|  | |  | Cohort | | Patients in cohort | Patients with outcome | Median survival (days) | Survival probability at end of time window | | |
|  | |  | 1 | CTLI + ENDOV | 3,727 | 480 | -- | 82.26% | | |
|  | |  | 2 | CLAUDICATION + ENDOV | 3,727 | 477 | -- | 82.85% | | |
|  | | | | | | | | | | |
|  | |  |  | | χ^2^ | df | p |  |  |  |
|  | |  | **Log-Rank Test** | | 0.952 | 1 | 0.329 |  |  |  |
|  | | | | | | | | | | |
|  | |  |  | | Hazard Ratio | 95% CI | χ^2^ | df | p | |
|  | |  | **Hazard Ratio and Proportionality** | | 1.065 | (0.938, 1.209) | 2.073 | 1 | 0.150 | |
|  | | | | | | | | | | |

## Supplementary Table S1. Comprehensive Code Dictionary

| Category | Description | Codes |
| --- | --- | --- |
| Cohort Definition | Peripheral artery disease (PAD) | ICD-10-CM: I70.2x, I70.3x, I70.4x, I70.5x, I70.6x |
|  | Claudication | ICD-10-CM: I70.21x |
|  | CLTI – rest pain | ICD-10-CM: I70.22x, I70.32x, I70.42x, I70.52x, I70.62x, I70.72x |
|  | CLTI – ulceration | ICD-10-CM: I70.23x, I70.24x, I70.33x–I70.43x, I70.53x–I70.63x, I70.73x |
|  | CLTI – gangrene | ICD-10-CM: I70.26x, I70.36x, I70.46x, I70.56x, I70.66x, I70.76x |
| Index Procedures |  |  |
|  | Endovascular revascularization – iliac | CPT: 37220–37223 |
|  | Endovascular revascularization – femoral/popliteal | CPT: 37224–37227 |
|  | Endovascular revascularization – tibial/peroneal | CPT: 37228–37231 |
|  | Peripheral thrombolysis | CPT: 37211–37214 |
|  | Peripheral arterial dilation/stenting | ICD-10-PCS: 047K3ZZ, 047L3ZZ, 047M3ZZ, 047N3ZZ, 02703ZZ |
| Exclusion Criteria |  |  |
|  | Major lower-extremity amputation | CPT: 27290, 27295, 27590–27592, 27598, 27880–27889 |
|  | Lower-extremity detachment | ICD-10-PCS: 0Y62xxx, 0Y67xxx, 0Y68xxx, 0Y6C–0Y6J |
|  | Acquired absence of lower extremity | ICD-10-CM: Z89.51x–Z89.62x |
|  | Surgical bypass revascularization | CPT: 35538–35571, 35666, 35681–35683 |
| Baseline Diagnoses |  |  |
|  | Hypertension | ICD-10-CM: I10–I15 |
|  | Coronary artery disease | ICD-10-CM: I25.x |
|  | Heart failure | ICD-10-CM: I50.x |
|  | Atrial fibrillation/flutter | ICD-10-CM: I48.x |
|  | Diabetes mellitus | ICD-10-CM: E10.x–E11.x |
|  | Chronic kidney disease | ICD-10-CM: N18.x |
|  | Essential (primary) hypertension | I10 |
|  | Diabetes mellitus | E08-E13 |
|  | Hyperlipidemia, unspecified | E78.5 |
|  | Other hyperlipidemia | E78.4 |
|  | Atherosclerotic heart disease of native coronary artery | I25.1 |
|  | Heart failure | I50 |
|  | Chronic kidney disease (CKD) | N18 |
|  | Chronic ischemic heart disease | I25 |
|  | Cerebral infarction | I63 |
|  | Old myocardial infarction | I25.2 |
|  | Atrial fibrillation and flutter | I48 |
|  | Nicotine dependence | F17 |
| Medications |  |  |
|  | aspirin | 1191 |
|  | clopidogrel | 32968 |
|  | prasugrel | 613391 |
|  | ticagrelor | 1116632 |
|  | cangrelor | 1656052 |
|  | atorvastatin | 83367 |
|  | rosuvastatin | 301542 |
|  | ACE INHIBITORS | CV800 |
|  | ANGIOTENSIN II INHIBITOR | CV805 |
|  | metoprolol | 6918 |
|  | carvedilol | 20352 |
|  | bisoprolol | 19484 |
|  | warfarin | 11289 |
|  | apixaban | 1364430 |
|  | rivaroxaban | 1114195 |
|  | edoxaban | 1599538 |
|  | dabigatran etexilate | 1037042 |
|  | Sodium-glucose co-transporter 2 (SGLT2) inhibitors | A10BK |
|  | Glucagon-like peptide-1 (GLP-1) analogues | A10BJ |

**Table S2: Definitions of Outcomes**

| MACE | | | | |
| --- | --- | --- | --- | --- |
|  | **Outcome definition** | | | |
|  | | Demographics | Deceased | Deceased |
|  | | Diagnosis | UMLS:ICD10CM:I21 | Acute myocardial infarction |
|  | | Diagnosis | UMLS:ICD10CM:I21.4 | Non-ST elevation (NSTEMI) myocardial infarction |
|  | | Diagnosis | UMLS:ICD10CM:I21.3 | ST elevation (STEMI) myocardial infarction of unspecified site |
|  | | Diagnosis | UMLS:ICD10CM:I21.9 | Acute myocardial infarction, unspecified |
|  | | Diagnosis | UMLS:ICD10CM:I21.A | Other type of myocardial infarction |
|  | | Diagnosis | UMLS:ICD10CM:I21.A1 | Myocardial infarction type 2 |
|  | | Diagnosis | UMLS:ICD10CM:I21.1 | ST elevation (STEMI) myocardial infarction of inferior wall |
|  | | Diagnosis | UMLS:ICD10CM:I21.0 | ST elevation (STEMI) myocardial infarction of anterior wall |
|  | | Diagnosis | UMLS:ICD10CM:I63 | Cerebral infarction |
|  | | Diagnosis | UMLS:ICD10CM:I63.50 | Cerebral infarction due to unspecified occlusion or stenosis of unspecified cerebral artery |
|  | **Settings for the performed analyses** | | | |
|  | | Kaplan - Meier survival analysis | | including patients with outcome prior to the time window |
|  | | Risk analysis | | including patients with outcome prior to the time window |
| All cause mortality | | | | |
|  | **Outcome definition** | | | |
|  | | Demographics | Deceased | Deceased |
|  | **Settings for the performed analyses** | | | |
|  | | Risk analysis | | including patients with outcome prior to the time window |
|  | | Kaplan - Meier survival analysis | | including patients with outcome prior to the time window |
| Stroke/TIA | | | | |
|  | **Outcome definition** | | | |
|  | | Diagnosis | UMLS:ICD10CM:I63 | Cerebral infarction |
|  | | Diagnosis | UMLS:ICD10CM:I63.50 | Cerebral infarction due to unspecified occlusion or stenosis of unspecified cerebral artery |
|  | | Diagnosis | UMLS:ICD10CM:G45 | Transient cerebral ischemic attacks and related syndromes |
|  | | Diagnosis | UMLS:ICD10CM:G45.9 | Transient cerebral ischemic attack, unspecified |
|  | **Settings for the performed analyses** | | | |
|  | | Kaplan - Meier survival analysis | | including patients with outcome prior to the time window |
|  | | Risk analysis | | including patients with outcome prior to the time window |
| heart failure hosp | | | | |
|  | **Outcome definition** | | | |
|  | | Diagnosis | UMLS:ICD10CM:I50.21 | Acute systolic (congestive) heart failure |
|  | | Diagnosis | UMLS:ICD10CM:I50.31 | Acute diastolic (congestive) heart failure |
|  | | Diagnosis | UMLS:ICD10CM:I50.811 | Acute right heart failure |
|  | | Diagnosis | UMLS:ICD10CM:I50.33 | Acute on chronic diastolic (congestive) heart failure |
|  | | Diagnosis | UMLS:ICD10CM:I50.23 | Acute on chronic systolic (congestive) heart failure |
|  | | Diagnosis | UMLS:ICD10CM:I50.813 | Acute on chronic right heart failure |
|  | | Diagnosis | UMLS:ICD10CM:I50.43 | Acute on chronic combined systolic (congestive) and diastolic (congestive) heart failure |
|  | | Diagnosis | UMLS:ICD10CM:I50.41 | Acute combined systolic (congestive) and diastolic (congestive) heart failure |
|  | **Settings for the performed analyses** | | | |
|  | | Kaplan - Meier survival analysis | | including patients with outcome prior to the time window |
|  | | Risk analysis | | including patients with outcome prior to the time window |
| inpatient hosp | | | | |
|  | **Outcome definition** | | | |
|  | | Visit | UMLS:HL7V3.0:VisitType:ACUTE | Visit: Inpatient Acute |
|  | **Settings for the performed analyses** | | | |
|  | | Kaplan - Meier survival analysis | | including patients with outcome prior to the time window |
|  | | Risk analysis | | including patients with outcome prior to the time window |
| AKI | | | | |
|  | **Outcome definition** | | | |
|  | | Diagnosis | UMLS:ICD10CM:N17 | Acute kidney failure |
|  | **Settings for the performed analyses** | | | |
|  | | Kaplan - Meier survival analysis | | including patients with outcome prior to the time window |
|  | | Risk analysis | | including patients with outcome prior to the time window |
| Repeat revasc | | | | |
|  | **Outcome definition** | | | |
|  | | Procedure | UMLS:CPT:37220 | Revascularization, endovascular, open or percutaneous, iliac artery, unilateral, initial vessel; with transluminal angioplasty |
|  | | Procedure | UMLS:CPT:37221 | Revascularization, endovascular, open or percutaneous, iliac artery, unilateral, initial vessel; with transluminal stent placement(s), includes angioplasty within the same vessel, when performed |
|  | | Procedure | UMLS:CPT:37222 | Revascularization, endovascular, open or percutaneous, iliac artery, each additional ipsilateral iliac vessel; with transluminal angioplasty (List separately in addition to code for primary procedure) |
|  | | Procedure | UMLS:CPT:37223 | Revascularization, endovascular, open or percutaneous, iliac artery, each additional ipsilateral iliac vessel; with transluminal stent placement(s), includes angioplasty within the same vessel, when performed (List separately in addition to code for primary procedure) |
|  | | Procedure | UMLS:CPT:37224 | Revascularization, endovascular, open or percutaneous, femoral, popliteal artery(s), unilateral; with transluminal angioplasty |
|  | | Procedure | UMLS:CPT:37225 | Revascularization, endovascular, open or percutaneous, femoral, popliteal artery(s), unilateral; with atherectomy, includes angioplasty within the same vessel, when performed |
|  | | Procedure | UMLS:CPT:37226 | Revascularization, endovascular, open or percutaneous, femoral, popliteal artery(s), unilateral; with transluminal stent placement(s), includes angioplasty within the same vessel, when performed |
|  | | Procedure | UMLS:CPT:37228 | Revascularization, endovascular, open or percutaneous, tibial, peroneal artery, unilateral, initial vessel; with transluminal angioplasty |
|  | | Procedure | UMLS:CPT:37229 | Revascularization, endovascular, open or percutaneous, tibial, peroneal artery, unilateral, initial vessel; with atherectomy, includes angioplasty within the same vessel, when performed |
|  | | Procedure | UMLS:CPT:37227 | Revascularization, endovascular, open or percutaneous, femoral, popliteal artery(s), unilateral; with transluminal stent placement(s) and atherectomy, includes angioplasty within the same vessel, when performed |
|  | | Procedure | UMLS:CPT:35538 | Bypass graft, with vein; aortobi-iliac |
|  | | Procedure | UMLS:CPT:35539 | Bypass graft, with vein; aortofemoral |
|  | | Procedure | UMLS:CPT:35540 | Bypass graft, with vein; aortobifemoral |
|  | | Procedure | UMLS:CPT:35556 | Bypass graft, with vein; femoral-popliteal |
|  | | Procedure | UMLS:CPT:35558 | Bypass graft, with vein; femoral-femoral |
|  | | Procedure | UMLS:CPT:35560 | Bypass graft, with vein; aortorenal |
|  | | Procedure | UMLS:CPT:35563 | Bypass graft, with vein; ilioiliac |
|  | | Procedure | UMLS:CPT:35565 | Bypass graft, with vein; iliofemoral |
|  | | Procedure | UMLS:CPT:35566 | Bypass graft, with vein; femoral-anterior tibial, posterior tibial, peroneal artery or other distal vessels |
|  | | Procedure | UMLS:CPT:35570 | Bypass graft, with vein; tibial-tibial, peroneal-tibial, or tibial/peroneal trunk-tibial |
|  | | Procedure | UMLS:CPT:35571 | Bypass graft, with vein; popliteal-tibial, -peroneal artery or other distal vessels |
|  | | Procedure | UMLS:CPT:35583 | In-situ vein bypass; femoral-popliteal |
|  | | Procedure | UMLS:CPT:35585 | In-situ vein bypass; femoral-anterior tibial, posterior tibial, or peroneal artery |
|  | | Procedure | UMLS:CPT:35587 | In-situ vein bypass; popliteal-tibial, peroneal |
|  | | Procedure | UMLS:CPT:35656 | Bypass graft, with other than vein; femoral-popliteal |
|  | | Procedure | UMLS:CPT:35661 | Bypass graft, with other than vein; femoral-femoral |
|  | | Procedure | UMLS:CPT:35663 | Bypass graft, with other than vein; ilioiliac |
|  | | Procedure | UMLS:CPT:35665 | Bypass graft, with other than vein; iliofemoral |
|  | | Procedure | UMLS:CPT:35666 | Bypass graft, with other than vein; femoral-anterior tibial, posterior tibial, or peroneal artery |
|  | | Procedure | UMLS:CPT:35682 | Bypass graft; autogenous composite, 2 segments of veins from 2 locations (List separately in addition to code for primary procedure) |
|  | | Procedure | UMLS:CPT:35683 | Bypass graft; autogenous composite, 3 or more segments of vein from 2 or more locations (List separately in addition to code for primary procedure) |
|  | **Settings for the performed analyses** | | | |
|  | | Kaplan - Meier survival analysis | | including patients with outcome prior to the time window |
|  | | Risk analysis | | including patients with outcome prior to the time window |
| Major bleed | | | | |
|  | **Outcome definition** | | | |
|  | | Diagnosis | UMLS:ICD10CM:I60.9 | Nontraumatic subarachnoid hemorrhage, unspecified |
|  | | Diagnosis | UMLS:ICD10CM:I61.9 | Nontraumatic intracerebral hemorrhage, unspecified |
|  | | Diagnosis | UMLS:ICD10CM:I62.9 | Nontraumatic intracranial hemorrhage, unspecified |
|  | | Diagnosis | UMLS:ICD10CM:K92.0 | Hematemesis |
|  | | Diagnosis | UMLS:ICD10CM:K92.1 | Melena |
|  | | Diagnosis | UMLS:ICD10CM:K92.2 | Gastrointestinal hemorrhage, unspecified |
|  | | Diagnosis | UMLS:ICD10CM:K25.0 | Acute gastric ulcer with hemorrhage |
|  | | Diagnosis | UMLS:ICD10CM:K26.0 | Acute duodenal ulcer with hemorrhage |
|  | | Diagnosis | UMLS:ICD10CM:E31.0 | Autoimmune polyglandular failure |
|  | | Diagnosis | UMLS:ICD10CM:I97.418 | Intraoperative hemorrhage and hematoma of a circulatory system organ or structure complicating other circulatory system procedure |
|  | | Diagnosis | UMLS:ICD10CM:I97.618 | Postprocedural hemorrhage of a circulatory system organ or structure following other circulatory system procedure |
|  | **Settings for the performed analyses** | | | |
|  | | Kaplan - Meier survival analysis | | including patients with outcome prior to the time window |
|  | | Risk analysis | | including patients with outcome prior to the time window |

**Table S3. One-Year Outcomes**

| **Outcome** | **CTLI + Endovascular, n (%)** | **Claudication + Endovascular, n (%)** | **Risk Difference (95% CI)** | **Risk Ratio (95% CI)** | **P-value** |
| --- | --- | --- | --- | --- | --- |
| MACE | 646/3,727 (17.3%) | 463/3,727 (12.4%) | 0.049 (0.033–0.065) | 1.40 (1.25–1.56) | <0.001 |
| All-cause mortality | 251/3,727 (6.7%) | 161/3,727 (4.3%) | 0.024 (0.014–0.035) | 1.56 (1.29–1.89) | <0.001 |
| Stroke/TIA | 256/3,727 (6.9%) | 220/3,727 (5.9%) | 0.010 (−0.001–0.021) | 1.16 (0.98–1.39) | 0.088 |
| Heart failure hospitalization | 259/3,727 (6.9%) | 211/3,727 (5.7%) | 0.013 (0.002–0.024) | 1.23 (1.03–1.46) | 0.022 |
| AKI | 510/3,727 (13.7%) | 373/3,727 (10.0%) | 0.037 (0.022–0.051) | 1.37 (1.21–1.55) | <0.001 |
| Repeat revascularization | 1,247/3,727 (33.5%) | 1,337/3,727 (35.9%) | −0.024 (−0.046–−0.003) | 0.93 (0.88–0.99) | 0.03 |
| Major bleeding | 296/3,727 (7.9%) | 227/3,727 (6.1%) | 0.019 (0.007–0.030) | 1.30 (1.10–1.54) | 0.002 |

##

## Table S4. Three-Year Outcomes

| **Outcome** | **CTLI + Endovascular, n (%)** | **Claudication + Endovascular, n (%)** | **Risk Difference (95% CI)** | **Risk Ratio (95% CI)** | **P-Value** |
| --- | --- | --- | --- | --- | --- |
| MACE | 1,032/3,727 (27.7%) | 884/3,727 (23.7%) | 0.040 (0.020–0.060) | 1.17 (1.08–1.26) | <0.001 |
| All-cause mortality | 491/3,727 (13.2%) | 386/3,727 (10.4%) | 0.028 (0.014–0.043) | 1.27 (1.12–1.44) | <0.001 |
| Stroke/TIA | 392/3,727 (10.5%) | 383/3,727 (10.3%) | 0.002 (−0.011–0.016) | 1.02 (0.90–1.17) | 0.733 |
| Heart failure hospitalization | 443/3,727 (11.9%) | 377/3,727 (10.1%) | 0.018 (0.004–0.032) | 1.18 (1.03–1.34) | 0.015 |
| AKI | 817/3,727 (21.9%) | 653/3,727 (17.5%) | 0.044 (0.026–0.062) | 1.25 (1.14–1.37) | <0.001 |
| Repeat revascularization | 1,601/3,727 (43.0%) | 1,774/3,727 (47.6%) | −0.046 (−0.069–−0.024) | 0.90 (0.86–0.95) | <0.001 |
| Major bleeding | 468/3,727 (12.6%) | 411/3,727 (11.0%) | 0.015 (0.001–0.030) | 1.14 (1.01–1.29) | 0.041 |

**Table S4. Five-Year Outcomes**

| **Outcome** | **CTLI + Endovascular,**  **n (%)** | **Claudication + Endovascular,**  **n (%)** | **Risk Difference (95% CI)** | **Risk Ratio (95% CI)** | **P-Value** |
| --- | --- | --- | --- | --- | --- |
| MACE | 1,301/3,727 (34.9%) | 1,146/3,727 (30.7%) | 0.042 (0.020–0.063) | 1.14 (1.06–1.21) | <0.001 |
| All-cause mortality | 698/3,727 (18.7%) | 582/3,727 (15.6%) | 0.031 (0.014–0.048) | 1.20 (1.09–1.33) | <0.001 |
| Stroke/TIA | 480/3,727 (12.9%) | 477/3,727 (12.8%) | 0.001 (−0.014–0.016) | 1.01 (0.89–1.13) | 0.917 |
| Heart failure hospitalization | 559/3,727 (15.0%) | 485/3,727 (13.0%) | 0.020 (0.004–0.036) | 1.15 (1.03–1.29) | 0.014 |
| AKI | 983/3,727 (26.4%) | 826/3,727 (22.2%) | 0.042 (0.023–0.062) | 1.19 (1.10–1.29) | <0.001 |
| Repeat revascularization | 1,711/3,727 (45.9%) | 1,910/3,727 (51.2%) | −0.053 (−0.076–−0.031) | 0.90 (0.86–0.94) | <0.001 |
| Major bleeding | 562/3,727 (15.1%) | 494/3,727 (13.3%) | 0.018 (0.002–0.034) | 1.14 (1.02–1.27) | 0.024 |
